# Supplementary material for: Microalga Nannochloropsis gaditana as a Sustainable Source of Bioactive Peptides: A Proteomic and In Silico Approach
Source: Foods. 2025 Jan 14;14(2):252. doi: 10.3390/foods14020252 (PMC11765504; doi:10.3390/foods14020252)
Supplement: Supplementary file 1 [file foods-14-00252-s001.zip › SupplementaryFigures_Paterson.pdf]

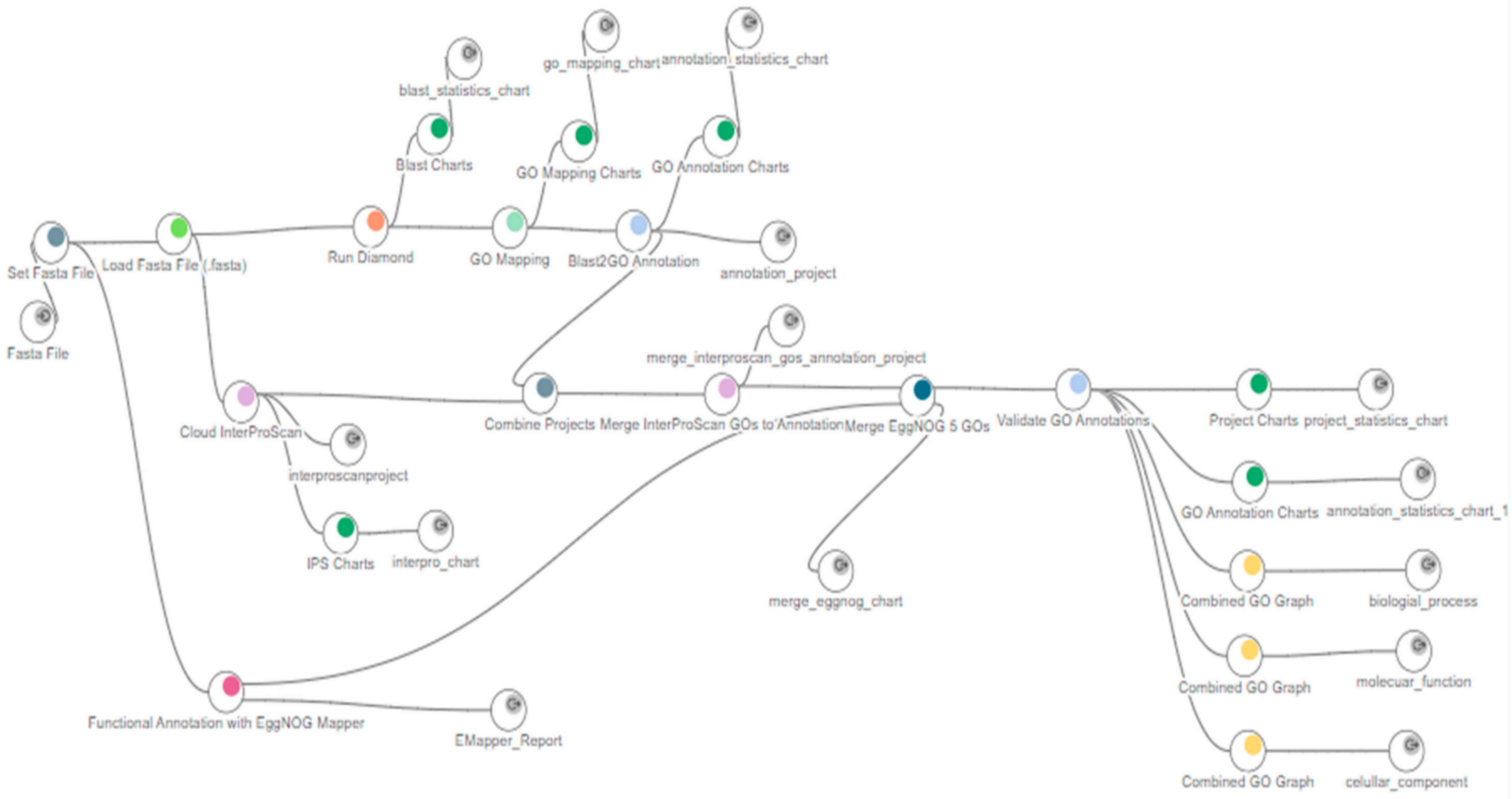

Supplementary Figure S1: Complete Omicbox workflow followed for the proteomic functional analysis of *Nannochloropsis gaditana*.

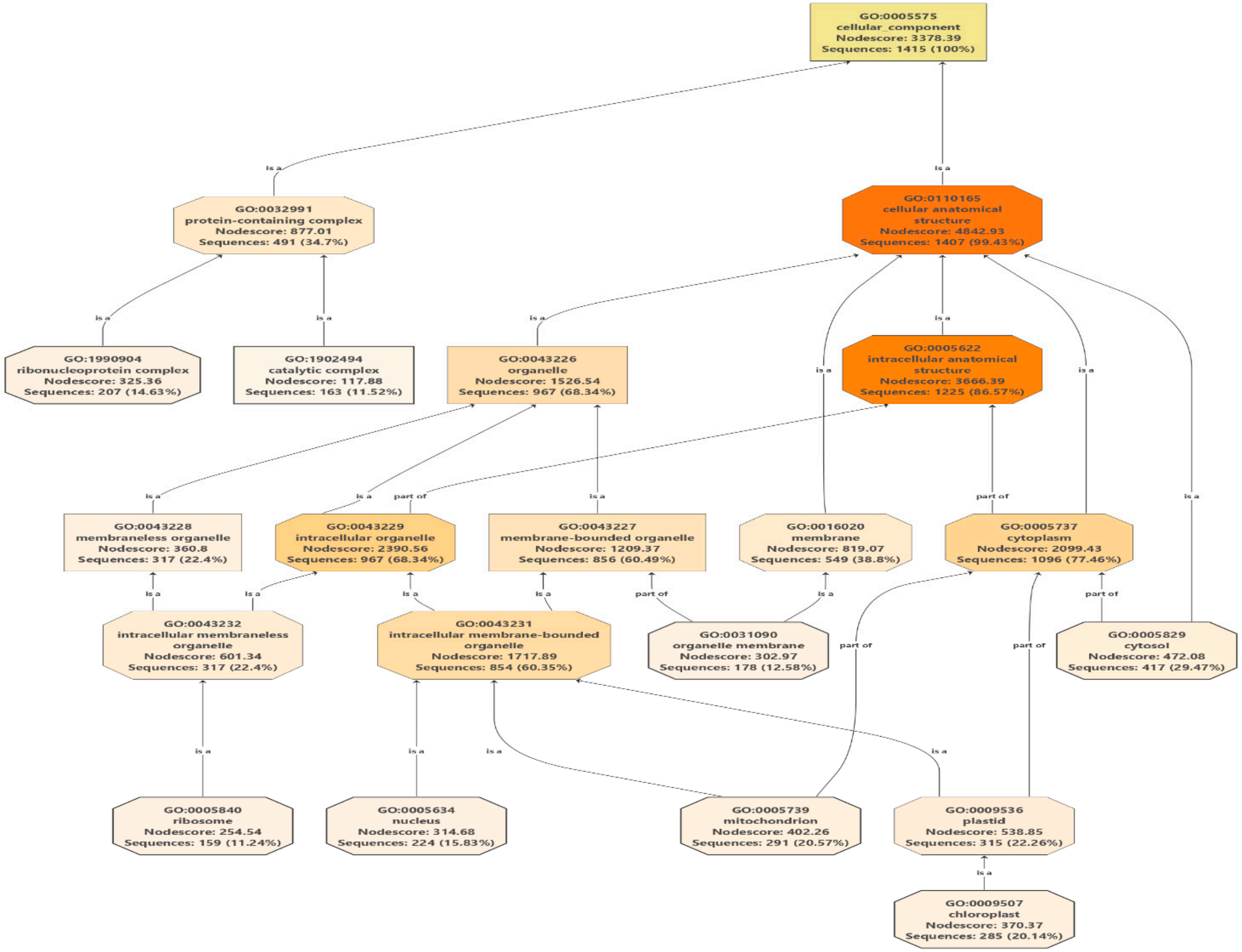

Supplementary Figure S2: Complete functional distribution of detected proteins from *Nannochloropsis gaditana* biomass in cellular component functional group using gene ontology (GO).

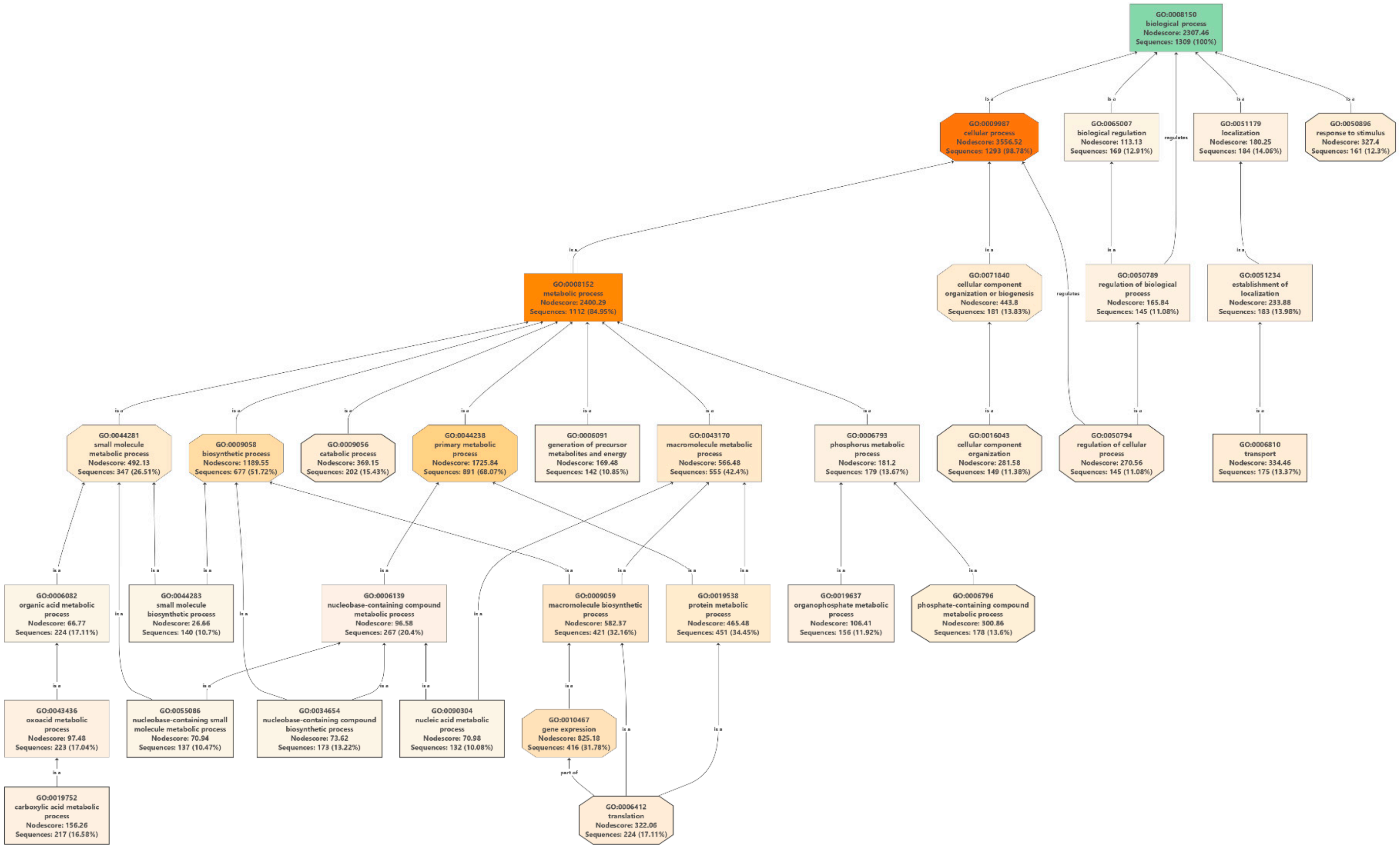

Supplementary Figure S3: Complete functional distribution of detected proteins from *Nannochloropsis gaditana* biomass in biological process functional group using gene ontology (GO).

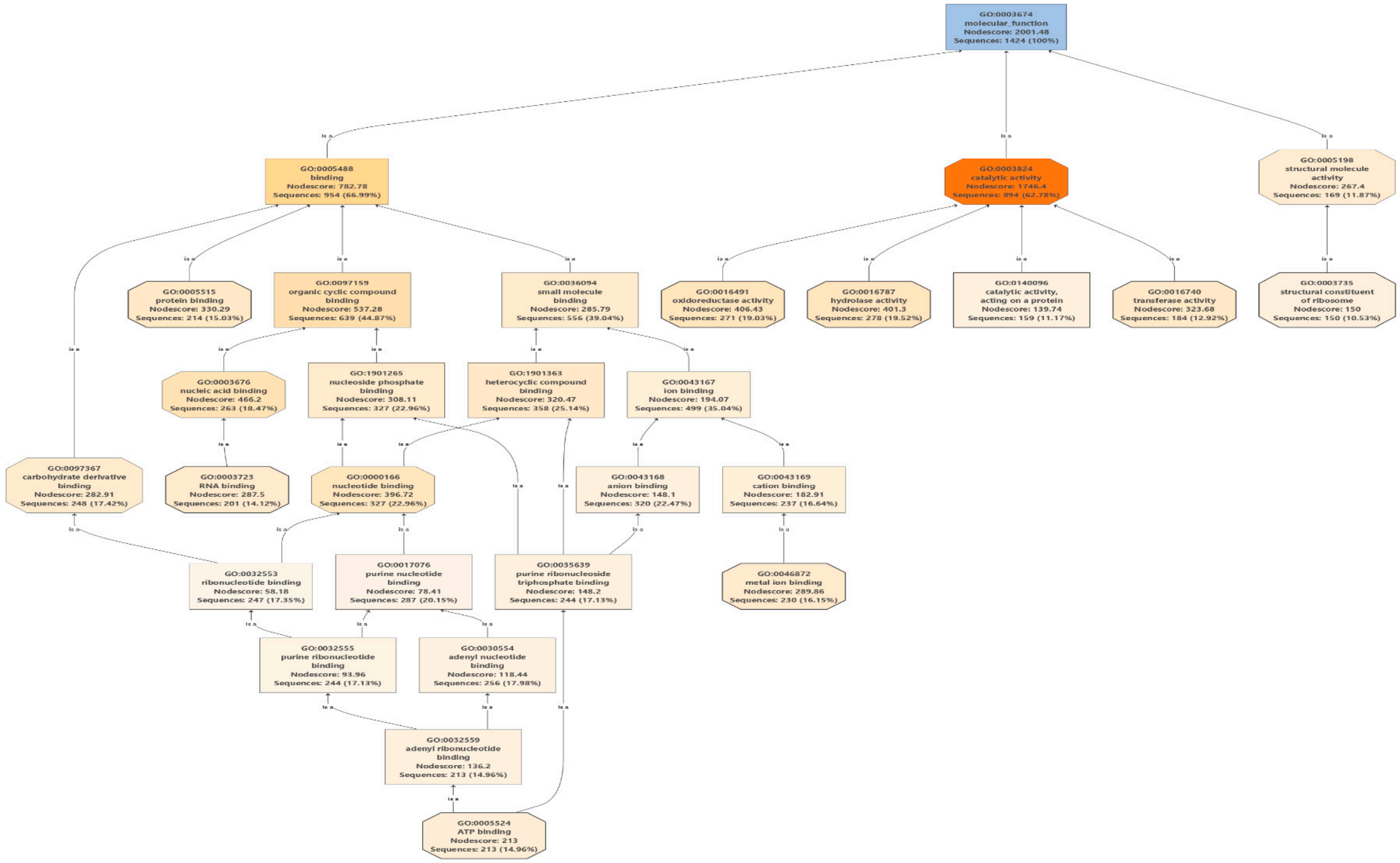

Supplementary Figure S4: Complete functional distribution of detected proteins from *Nannochloropsis gaditana* biomass in molecular function functional group using gene ontology (GO).
